# Behavioral paradigm for the evaluation of stimulation-evoked somatosensory perception thresholds in rats

**DOI:** 10.3389/fnins.2023.1202258

**Published:** 2023-06-13

**Authors:** Thomas J. Smith, Yupeng Wu, Claire Cheon, Arlin A. Khan, Hari Srinivasan, Jeffrey R. Capadona, Stuart F. Cogan, Joseph J. Pancrazio, Crystal T. Engineer, Ana G. Hernandez-Reynoso

**Affiliations:** ^1^School of Behavioral and Brain Sciences, The University of Texas at Dallas, Richardson, TX, United States; ^2^Department of Materials Science and Engineering, The University of Texas at Dallas, Richardson, TX, United States; ^3^Department of Bioengineering, The University of Texas at Dallas, Richardson, TX, United States; ^4^Department of Biomedical Engineering, Case Western Reserve University, Cleveland, OH, United States; ^5^Advanced Platform Technology Center, Louis Stokes Cleveland Veterans Affairs Medical Center, Cleveland, OH, United States; ^6^Texas Biomedical Device Center, The University of Texas at Dallas, Richardson, TX, United States

**Keywords:** intracortical microstimulation, somatosensory cortex, behavioral paradigm, microelectrode array, brain–machine interfaces

## Abstract

Intracortical microstimulation (ICMS) of the somatosensory cortex via penetrating microelectrode arrays (MEAs) can evoke cutaneous and proprioceptive sensations for restoration of perception in individuals with spinal cord injuries. However, ICMS current amplitudes needed to evoke these sensory percepts tend to change over time following implantation. Animal models have been used to investigate the mechanisms by which these changes occur and aid in the development of new engineering strategies to mitigate such changes. Non-human primates are commonly the animal of choice for investigating ICMS, but ethical concerns exist regarding their use. Rodents are a preferred animal model due to their availability, affordability, and ease of handling, but there are limited choices of behavioral tasks for investigating ICMS. In this study, we investigated the application of an innovative behavioral go/no-go paradigm capable of estimating ICMS-evoked sensory perception thresholds in freely moving rats. We divided animals into two groups, one receiving ICMS and a control group receiving auditory tones. Then, we trained the animals to nose-poke – a well-established behavioral task for rats – following either a suprathreshold ICMS current-controlled pulse train or frequency-controlled auditory tone. Animals received a sugar pellet reward when nose-poking correctly. When nose-poking incorrectly, animals received a mild air puff. After animals became proficient in this task, as defined by accuracy, precision, and other performance metrics, they continued to the next phase for perception threshold detection, where we varied the ICMS amplitude using a modified staircase method. Finally, we used non-linear regression to estimate perception thresholds. Results indicated that our behavioral protocol could estimate ICMS perception thresholds based on ~95% accuracy of rat nose-poke responses to the conditioned stimulus. This behavioral paradigm provides a robust methodology for evaluating stimulation-evoked somatosensory percepts in rats comparable to the evaluation of auditory percepts. In future studies, this validated methodology can be used to study the performance of novel MEA device technologies on ICMS-evoked perception threshold stability using freely moving rats or to investigate information processing principles in neural circuits related to sensory perception discrimination.

## 1. Introduction

Intracortical microstimulation (ICMS) of the somatosensory cortex via microelectrode arrays (MEAs) has been successfully used to evoke cutaneous and proprioceptive sensations in amputees and individuals with spinal cord injuries (Armenta Salas et al., 2018; Page et al., 2021; Bjånes et al., 2022; Christie et al., 2022). These sensations can provide somatosensory feedback for closed-loop brain-machine interfaces and neuroprosthetics (Carè et al., 2022), which has been demonstrated to improve the control of robotic arms (Flesher et al., 2021). However, once implanted into the brain, achieving long-term stability of perception thresholds with these devices has been challenging (Callier et al., 2015; Hughes et al., 2021; Urdaneta et al., 2022) due to multifactorial failure of the interface. These failures include surpassing the safety limits of electrical microstimulation (Shannon, 1992; Pancrazio et al., 2017; Kramer et al., 2019), foreign body response that can isolate the MEAs from the surrounding neural tissue (Rajan et al., 2015), neuroinflammation that leads to neuronal loss (Potter et al., 2012; Ereifej et al., 2018), and material cracking and delamination (Barrese et al., 2013). Despite the promises of using ICMS to restore sensation, these failure modes pose a barrier for more widespread use. Because of this, research to improve the long-term reliability of ICMS is needed. The majority of pre-clinical studies investigating ICMS involve non-human primates; however, ethical concerns and costs limit their use (Pankevich, 2012; Bailey and Taylor, 2016; Carvalho et al., 2019). Rodents have been widely used to investigate the recording performance of MEAs due to their availability, affordability, and ease of handling (Koivuniemi A. S. et al., 2011; El-Ayache and Galligan, 2020). However, the use of this model organism for evaluating ICMS-induced somatosensory perceptions has been hindered by the limited behavioral paradigms available for this purpose.

To our knowledge, three behavioral paradigms have been described in the literature for assessing ICMS in the primary somatosensory cortex of rodents (Koivuniemi A. et al., 2011; Öztürk et al., 2019; Urdaneta et al., 2021; Lycke et al., 2023). These behavioral tasks use either a freely moving passive avoidance psychophysical detection task, a freely moving active avoidance conditioning paradigm, or a head-fixed go/no-go task. All were successful at detecting thresholds for up to 33 weeks with 70–95% accuracy; however, all three paradigms involve water deprivation for up to 36 h prior to behavioral testing (Koivuniemi A. et al., 2011; Öztürk et al., 2019) which can produce stress (Vasilev et al., 2021) and confound chronic assessments. Alternative behavioral paradigms that use food-restriction have been described for the testing of auditory thresholds. An example of this is the well-established nose-poke behavioral paradigm (Schindler et al., 1993; Abolafia et al., 2011; Riley et al., 2021), a behavioral paradigm where a food-deprived rat is introduced into an operant conditioning chamber and trained to nose-poke through a hole on a side wall upon presentation of an auditory tone followed by a sugar pellet reward. While this behavioral task has been shown to be highly accurate with ~90% discrimination accuracy scores (Sloan et al., 2009; Riley et al., 2021) and effective for auditory psychophysical testing, it has not been used to assess ICMS-induced somatosensory perceptions because no adaptations of the task have been made to suit this need.

Here we describe an innovative operant conditioning behavioral task to effectively assess ICMS-evoked sensory perception thresholds. We adapted the well-established and validated nose-poke auditory task into a food positive reinforcement go/no-go behavioral paradigm in food-deprived, freely moving rats with a mild passive avoidance positive punishment – a behavioral approach in which an aversive stimulus is presented to decrease the likelihood of an undesired response (Casey et al., 2021) – air-puff. We implanted MEAs into Sprague–Dawley rats, targeting the forelimb area of the left primary somatosensory cortex (S1FL) and delivered electrical stimulation to modulate the neural activity and evoke artificial sensory percepts. We compared the accuracy of this task for ICMS perception thresholds with the accuracy of auditory tone discrimination for validation of the novel behavioral paradigm. Our results show that this behavioral protocol could estimate ICMS perception thresholds based on ~95% accuracy of all rat nose-poke responses to the conditioned stimulus, validating its use for future ICMS perception threshold investigations.

## 2. Materials and methods

### 2.1. Ethics statement

All animal handling, housing and procedures were approved by The University of Texas at Dallas IACUC (protocol #21–15) and in accordance with ARRIVE guidelines.

### 2.2. Animal use

We used six (*N* = 6) male Sprague–Dawley rats (Charles River Laboratories Inc., Houston, TX, US) that were single-housed in standard home cages under a reverse 12-h day/night cycle. We food-deprived the animals four consecutive days per week to a 90% free-feeding level that was redefined weekly to promote consistent performance during the behavioral task (Schindler et al., 1993) and given *ad libitum* access to food three consecutive days per week. Their weight was recorded on the last day of the week with *ad libitum* access to food, and before every behavioral session during the four consecutive days of food deprivation to assess welfare of the animal. If the weight before the behavioral session was below 90% of its recorded control weight, we provided supplemental rodent feed pellets to provide additional nourishment and excluded the animal from behavioral experimentation until the 90% free-feeding control weight was restored. Animals were given dustless reward pellets (F0021, Bio-Serv, Flemington, NJ, US) as positive reinforcement for the behavioral paradigm. These pellets contain a balanced caloric profile enriched with amino acids, carbohydrates, fatty acids, vitamins, and mineral mix to ensure the nutritional wellbeing of the animals despite food deprivation. In addition, we provided rats with supplemental regular food pellets (5LL2 - Prolab® RMH 1800, LabDiet, St. Louis, MO, US) after each behavioral session to maintain weight. This supplemental feed was calculated based on the number of reward pellets eaten during each behavioral session. Animals had *ad libitum* access to water at all times while in their standard home cages.

**Figure 1:**
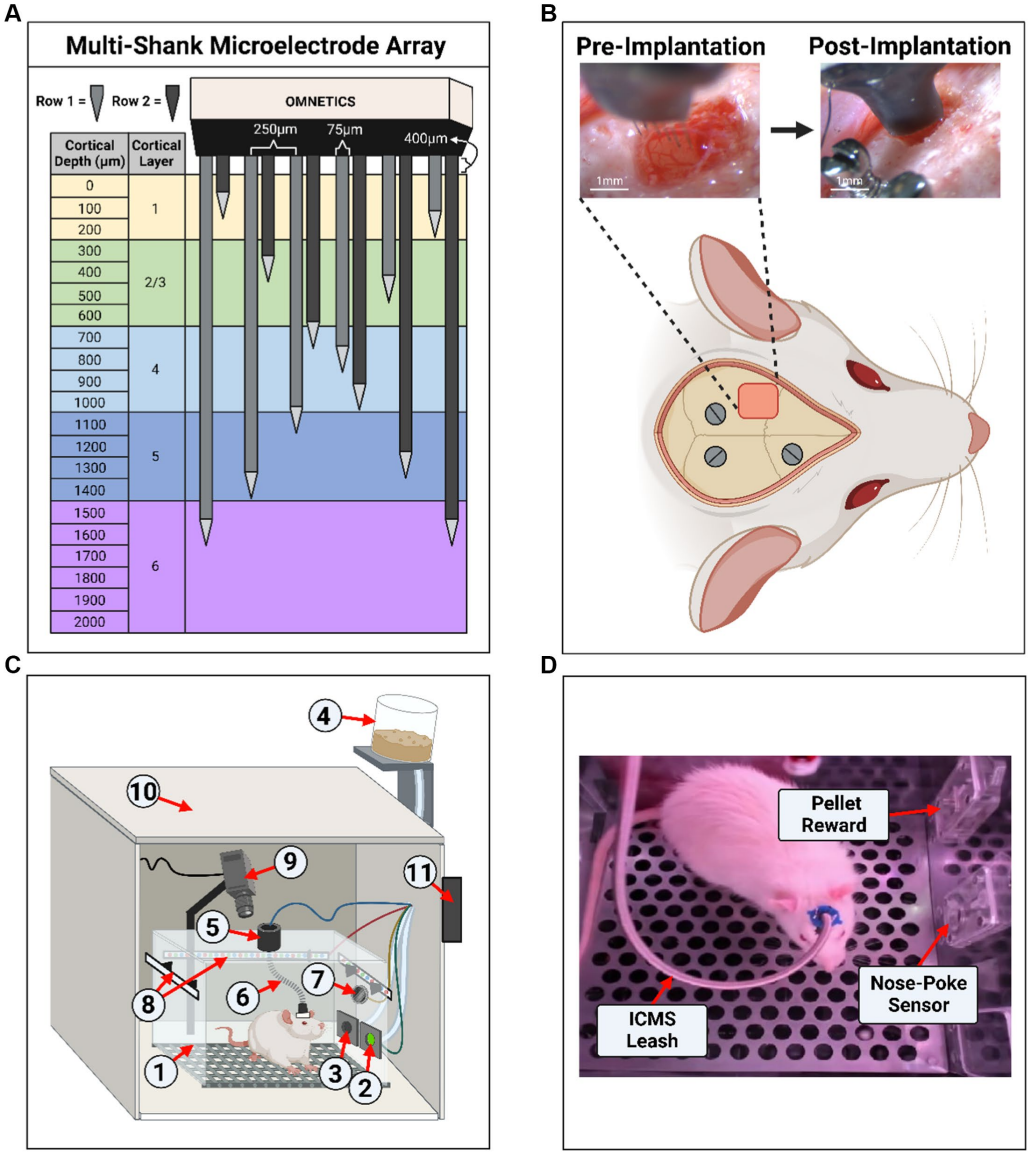
Experimental setup and microelectrode array implantation. **(A)** Diagram of the 12-shank MEA with opposing slanted rows penetrating all layers of the somatosensory cortex. **(B)** Example of an implantation surgery (craniotomy, durotomy, and microelectrode array insertion) within the left primary somatosensory cortex, forelimb area (S1FL). Three stainless steel screws were inserted into the skull for ground/counter electrodes and headcap anchors. **(C)** Illustration of the operant conditioning chamber setup used for animal behavior. The setup contains: (1) operant conditioning chamber, (2) nose-poke sensor hole, (3) sugar pellet reward hole, (4) pellet dispenser, (5) commutator, (6) ICMS leash, (7) speaker, (8) RGB LED strips, (9) webcam/camera, (10) noise reduction chamber, (11) microcontroller board hub. **(D)** Screenshot from a behavioral live stream session depicting a real-world view. In the image, the sugar pellet reward hole, nose-poke sensor hole, and the ICMS leash were shown.

Rats were randomized and divided into two groups. The first was the experimental group, which received implantation with a multi-shank MEA (MEA-PI-A3-00-12-0.01-[1–2]-3–0.25-0.25-1-1SS; Microprobes for Life Science, Gaithersburg, MD, US) consisting of 12 Pt/Ir (70% Pt, 30% Ir, 0.01 MΩ) microwires of 75 μm diameter, insulated with polyamide. The tips of each microwire had an exposed geometric surface area ranging between 6,000 and 9,000 μm^2^. The MEA design has two rows of six microwires each, which slant in opposing directions ranging in length between 0.5–2 mm (Figure 1A). Selection of this device was based on robustness and demonstrated ability to stimulate neural tissue (Miyamoto et al., 2017). The two rows with slanted opposing directions were designed to target multiple layers, which may have different threshold levels (Urdaneta et al., 2021). In addition, this design has the ability to activate a large volume of tissue, which ensures the generation of sensory percepts for the development of the protocol described here and has been demonstrated to generate more natural percepts in humans (Bjånes et al., 2022). Each MEA includes an additional 2 mm microwire that serves as the reference electrode. The experimental group received ICMS (*n* = 3) during the behavioral task. The second group was a control group (*n* = 3), which underwent sham surgery and received auditory tones during the behavioral task. The sham surgery consisted of a craniotomy and durotomy procedure comparable with the experimental group without implantation of the MEA. The goal of the control group was to compare the accuracy of the behavioral paradigm presented here. The operant chamber apparatus was thoroughly cleaned with a 70% ethanol solution between each session to help eliminate any distracting scents between animal subjects. After completing the behavioral testing, animals in both groups were subjected to the same behavioral task without electrical stimulation. This was done to act as an intragroup negative control to validate the ICMS or audio tone, as the only interpreted conditioning cue by verifying changes in accuracy during the absence of a stimulus.

### 2.3. Surgical procedure

Rats underwent a surgical procedure for sham and MEA implantation as previously described (Sturgill et al., 2022). Briefly, animals were anesthetized using vaporized isoflurane (1.8–2.5%) mixture with medical grade oxygen (500 ml/min, SomnoSuite® for Mice & Rats, Kent Scientific Corporation, Torrington, CT, US). The surgical team monitored vital signs throughout the surgical procedure while body temperature was maintained using a controlled far-infrared warming pad (PhysioSuite® for Mice & Rats, Kent Scientific Corporation, Torrington, CT, US). The scalp was shaved and animals were mounted onto a digital stereotaxic frame (David Kopf Instruments, Tujunga, CA, US). The skin at the surgical site was cleaned using three alternating applications of betadine and alcohol wipes. A subcutaneous injection of 0.5% bupivacaine hydrochloride (Marcaine, Hospira, Lake Forest, IL, USA) was given at the intended incision site. An incision was made through the midline of the scalp, muscles, and connective tissue. Next, the skull was leveled and centered in the stereotaxic frame using bregma, lambda, and the sagittal suture as references (± 0.1 mm). Three holes were then drilled into the skull to insert stainless-steel bone screws (Stoelting Co., Wood Dale, IL, USA) (Figure 1B). Then, a 2 mm x 3 mm craniotomy was made targeting the S1FL (AP: −0.5 mm, ML: 4 mm), followed by a durotomy (Figure 1B). The surgeon secured the ground wire to one of the mounted bone screws and implanted the MEA to a cortical depth of ~1.6 mm using a precision-controlled inserter (NeuralGlider, Actuated Medical, Inc., Ann Arbor, MI, USA) (Figure 1B). Implantation within the cranial window was done to avoid disruption of major surface blood vessels (Kozai et al., 2010; He et al., 2022). The implant site was then sealed with a biocompatible, transparent silicone elastomer adhesive (Kwik-Sil, World Precision Instruments, Sarasota, FL, USA), followed by a dental cement head cap to tether the MEA to the skull while also reducing the likelihood of contamination and infection. Then, the incision was closed using surgical staples and tissue adhesive (GLUture, World Precision Instruments, Sarasota, FL, USA). At the end of the surgical procedure, we injected each animal with 0.05 mL/kg intramuscular cefazolin (Med-Vet International, Mettawa, IL, USA) for antibiotic prophylaxis together with topical application triple-antibiotic ointment around the incision site. For analgesia, we administered either 0.15 mL/kg of subcutaneous slow-release (Buprenorphine SR-LAB, ZooPharm, LLC., Laramie, WY, USA) or 0.5 mL/kg of extended-release (Ethiqa XR, Fidelis Animal Health, North Brunswick, NJ, USA) buprenorphine depending on availability of the substance. When necessary, we administered a dose of buprenorphine after 72 h post-surgery if the animal showed signs of pain. Lastly, we provided sulfamethoxazole and trimethoprim oral suspension (200 mg/40 mg/5 ml, Aurobindo Pharma, Dayton, NJ, USA) in the animals’ drinking water (1 ml/100 ml drinking water) as an additional antibiotic for 7 days post-surgery.

### 2.4. Behavioral operant chamber, equipment and software

Figures 1C,D illustrates the behavioral operant chamber used for this study. The go/no-go behavioral paradigm was conducted within a commercially available operant conditioning chamber (OmniTrak, Vulintus, Inc., Lafayette, CO, USA). This chamber had two holes in one of the side walls, one containing an infrared break-beam sensor (nose-poke sensor) and a second hole connected to a precision pellet dispenser. In addition, the nose-poke hole had the capability of delivering a mild air-puff from a medical-grade compressed air cylinder tube as positive punishment. This air-puff was controlled via a pneumatic solenoid (SKUSKD1384729, AOMAG) connected to an Inland Nano microcontroller through a relay switch to deliver air to the nose-poke sensor hole. A rotating commutator (76-SR-12, NTE Electronics, Bloomfield, NJ, USA) was bolted at the top of the operant chamber to allow the animals to roam free while connected to an external stimulator (PlexStim, Plexon Inc., Dallas, TX, USA) for ICMS. A custom cord was designed to connect the animal to the commutator for ICMS, incorporating an Omnetics (A79021-001, Omnetics Connector Corporation, Minneapolis, MN, USA) adapter and surrounded with a stainless-steel spring cable shielding (#6Y000123101F, Protech International Inc., Boerne, TX, USA) to protect the wires against biting. For the auditory control group, auditory tones were presented through a mini speaker (Product ID: 3923, Adafruit Industries, New York City, NY, USA) that was placed inside the chamber and connected to a PC’s headphone auxiliary port. The chamber was illuminated via an RGB LED strip controlled by the Inland Nano microcontroller. A webcam (960–001105, Logitech, Lausanne, CH, USA) was mounted to the chamber to record a live video stream of the animal during behavioral sessions. Finally, the chamber was enclosed inside a sound-reduction chamber equipped with a fan for cooling and air circulation. All modules were connected and controlled by an ATMEGA2560 microcontroller board hub (OmniTrak Controller V3.0, Vulintus Inc., Lafayette, CO, USA), interfaced using custom MATLAB (R2022b, Mathworks, Natick, MA, USA) software. The RBG LED strip and solenoid valve required a supplemental 12 V 2A DC power supply to power the devices.

In addition, we developed a custom MATLAB GUI application (Supplementary Figure S1) that simultaneously controls and displays the behavioral task parameters, monitors animal performance, and records session data. While a behavioral session is active, the application feeds the session video live stream from the operant chamber to the researcher, as shown in Supplementary Figure S1. Furthermore, this GUI included specialized buttons for the researcher to annotate instances during each session where we deemed the animals distracted (e.g., grooming or turning away from the sensors/modules for the entire trial duration) for exclusion from analysis. After each session, a second researcher validated the annotations offline to reduce bias. Additional features of the GUI application include a button for manually dispensing sugar pellets, the ability to record voltage transients throughout the session, and the capability to choose which electrode channels are delivered ICMS. This custom MATLAB and UI/UX behavior software is available as an open-source package on GitHub.^1^

### 2.5. Electrical stimulation and auditory parameters

Electrical stimulation for ICMS was delivered to 10 electrode sites simultaneously per implanted MEA. The stimulation parameters selected for this work were previously established by another group and validated to evoke somatosensory percepts in rats (Urdaneta et al., 2021). We used current-controlled, charge-balanced symmetric biphasic waveforms with a cathodal-leading phase, a frequency of 320 Hz, pulse width of 200 μs per phase, 40 μs interphase interval, with a 650 ms train duration (PlexStim, Plexon Inc., Dallas, TX, USA). Current amplitudes used in this work ranged from 0 to 25 μA corresponding to a charge of 0–5 nC/ph. The maximum charge limit set for all experiments was 5 nC/ph per electrode stimulated simultaneously across 10 channels. Seven to twelve days after implantation but before operant conditioning training, we estimated a provisional ICMS naïve perception threshold for each animal by slowly increasing the charge/phase across all 10 individually pulsed channels simultaneously from 0 to 5 nC/ph until a physical response (e.g., paw withdrawal) was observed. Once this provisional perception threshold was determined, this naïve perception threshold was subsequently used as the starting known threshold for the go/no-go behavioral paradigm.

For the auditory control group, auditory tone parameters were derived from prior go/no-go paradigms (Green et al., 1979; Engineer et al., 2008; Sloan et al., 2009). In our experiment, we used a carrier frequency of 6 kHz pure tone sinusoidal wave with a 100 kHz sampling rate, 500 ms tone duration, and a 50 ms beginning/end tone ramp duration. Using a sound level meter (Extech Instruments, Nashua, NH, US), the produced output intensity of this auditory training tone was measured to be ~90 dB in reference to the sound pressure level (SPL) of 0 dB, which is the intensity of sound waves relative to the minimum threshold of human hearing.

### 2.6. Go/no-go behavioral training

**Figure 2:**
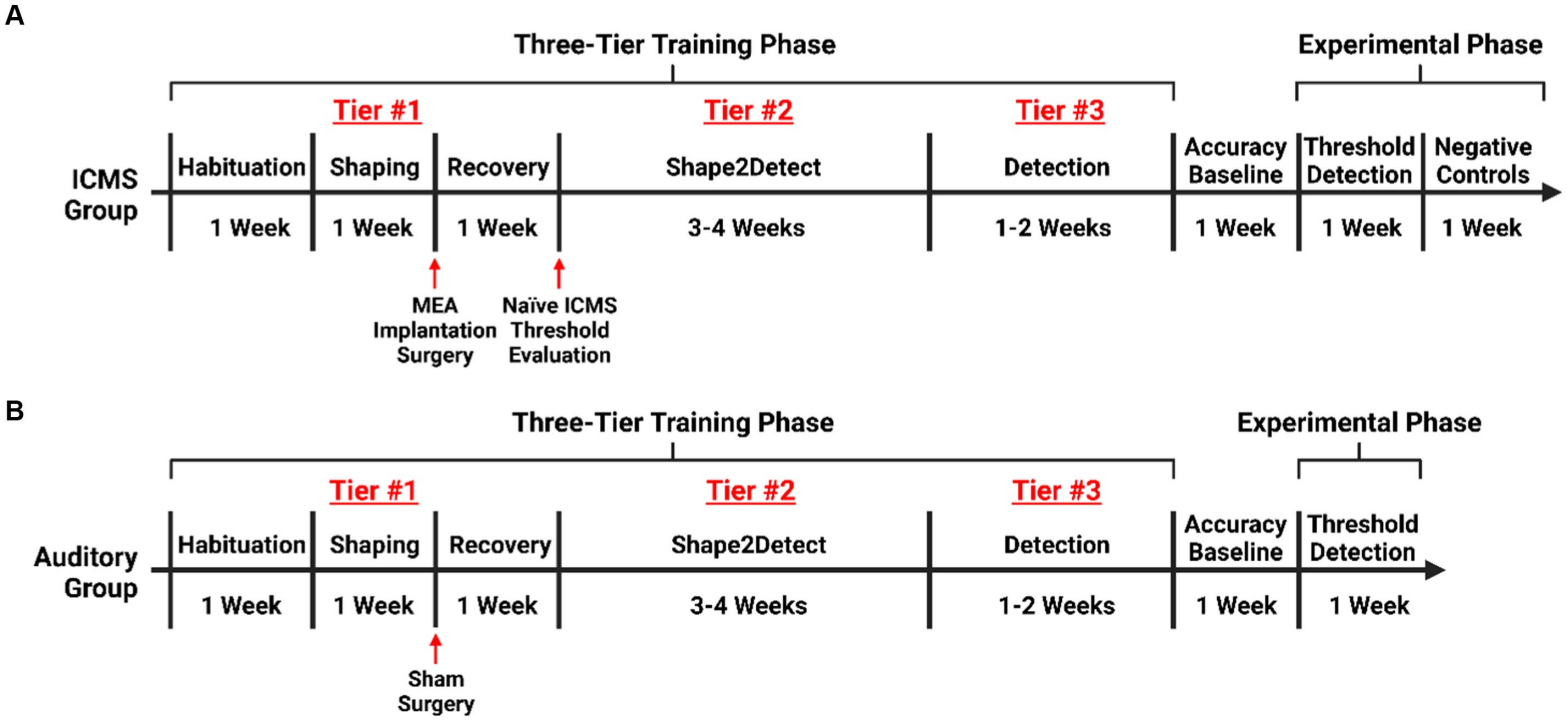
Experimental timeline. Timeline for training rats on the go/no-go behavioral paradigm. **(A)** Training for rats in the ICMS experimental group with an extended phase where no ICMS is presented, acting as an intragroup negative control. **(B)** Training for rats in the auditory control.

**Figure 3:**
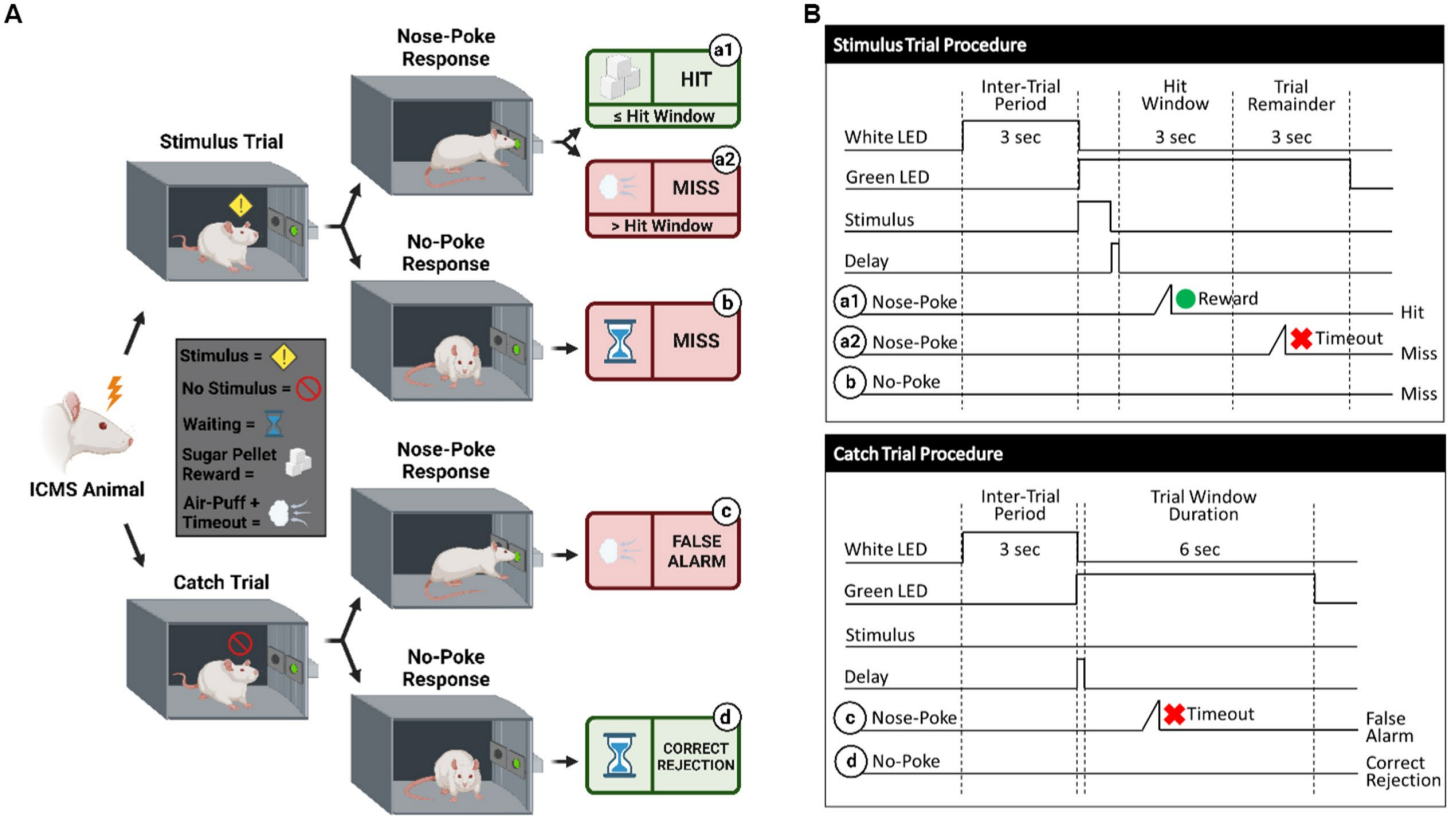
Behavioral paradigm for go/no-go task. **(A)** Visualization of the go/no-go behavioral paradigm with possible responses to ICMS. **(B)** Illustration of the go/no-go behavioral paradigm outlining trial types. Schematic shows differences between the stimulus trials (top) and the catch trials (bottom). Depending on the response to the presented trial type, the animal can either receive a sugar pellet reward (hit) symbolized by the green circle, an 8 s timeout sequence + air puff (false alarm) symbolized by the red x, or nothing (miss/correct rejection). A 150 ms delay immediately following a stimulus presentation is used, where the nose-poke sensor does not trigger.

We trained rats on the go/no-go behavioral paradigm following a three-tier protocol. Namely, Shaping, Shape2Detect, and Detection, as shown in Figure 2. Each tier is designed to gradually train every animal to nose-poke following a presented stimulus (ICMS or auditory tone) to receive a reward pellet in the go/no-go paradigm as shown in Figure 3A. Before training began, animals were habituated for a minimum of 10 h until the animal tolerated handling and head restraint for at least two consecutive minutes. This habituation allowed for manipulation of the animals and connection of the implanted MEA to the rotating commutator hardware before each behavioral session. During the habituation period, the animals were fed reward pellets to incentivize the reward-seeking behavior.

### 2.7. First tier: shaping

Shaping was the first tier for the go/no-go training, which consisted of 1-h sessions, 5 days per week. The goal of this phase was to train the animal on the nose-poke behavior task via positive reinforcement. First, the animal was introduced into the operant chamber and allowed to freely roam. The chamber was illuminated with white light via the RGB LED strip. After 3 s, the RGB LED strip was configured to illuminate with green light for an indefinite amount of time, indicating a trial had begun. A pellet reward was dispensed when the animal nose-poked through the nose-poke hole as a positive-reinforcement to promote this behavior unless the animal poked within the first 150 ms of the trial. This delay was incorporated to prevent accidental nose-pokes from occurring at the start of a trial. After the animal nose-poked, the green light turned back to white light for an inter-trial period of 3 s. If the animal nose-poked during the inter-trial period, no reward pellet was dispensed. When needed, we manually dispensed pellets when animals approached the nose-poke hole, even if the animal did not poke to encourage exploration. Animals were considered proficient in the Shaping task when they received 100+ reward pellets for two consecutive sessions without manual pellets dispensed. After passing this tier, they received either surgery for MEA implantation, or sham surgery. If a rat did not meet the 100+ pellet reward within 10 sessions, the animal was excluded from the study.

### 2.8. Second tier: Shape2Detect

Shape2Detect was the second tier for the go/no-go task training, as shown in Figure 2. During this phase, animals were trained to nose-poke only upon presentation of either the ICMS at their pre-established naïve threshold or the auditory training tone at ~90 dB SPL, depending on their group allocation. We began each session by placing the animal into the apparatus once per day, 4 days per week for 60-min-long sessions. At the start of each session, the operant chamber was illuminated by white light from the RGB LED strip. When each trial began, the RGB LED strip changed to green light to indicate the beginning of a trial (Figure 3B). During this phase, animals were presented with two types of trials: stimulus trials or catch trials as outlined in Figures 3A,B. A stimulus trial was defined as the presentation of the ICMS or auditory tone, whereas a catch trial consisted of an absence of stimulation or sound. Stimulus and catch trials were presented during a trial window duration that was followed by a 3 sinter-trial period of white light. The trial window duration varied as time progressed throughout the session, as shown in Table 1. For the first 20 min, the trial window duration was set to 3 s. The next 10 min had trial durations of 4 s, the following 10 min durations of 5 s, and the final 10 min durations of 6 s. Throughout the session, the likelihood of a stimulus trial being presented versus a catch trial was varied. The first 10 min had an 83.3% probability of presenting a stimulus trial (with a 16.7% probability of catch trials) and then changed until the last 10 min had a 50% probability of presenting a stimulus trial (50% probability of catch trials). The rationale for varying this probability was to increase the frequency of stimulus exposure at the beginning of the session, providing the animal ample opportunities to associate the stimulus presentation with a reward. Then, we decreased the frequency of the stimulus exposure as the session progressed to avert continuous poking and encourage discriminatory decision making. Finally, hit windows and timeouts were also varied throughout the session (see Table 1). Hit windows were defined as the duration of time after the presentation of a stimulus during which the animal can nose-poke and receive a pellet reward, while timeouts were characterized as a period of red-light illumination in the apparatus (Figure 3B). A hit was determined if an animal nose-poked during the hit window. If an animal nose-poked after the hit window (trial remainder) or during a catch trial, it received a mild-air puff as a positive punishment and triggered a timeout period. The first instance was classified as a miss for quantification purposes; the latter as a false alarm. to reinforce the rat’s ability to ignore trials in the absence of stimulus and discourage nose-poking freely. In continuation, if the animal poked during the timeout period, it received an additional air-puff with more time added to the timeout. The pressure of the air-puff was adjusted as needed so that it was enough to prevent timeouts but not to completely deter the animal from nose-poking. Furthermore, if the animal failed to nose-poke for 10 stimulus trials in a row, the session would be paused and resumed only after the animal nose-poked again. Finally, a correct rejection was defined as the animal refraining from nose-poking during a catch trial.

**Table 1:** Shape2Detect behavioral training task parameters.

Session time (min)	Trial window duration (s)	Stimulus trial probability (%)	Hit window (s)	Timeout (s)
0–9	3	83.3	3	2
10–19	3	71.4	3	3
20–29	4	66.7	4	3
30–39	5	60.0	5	5
40–60	6	50.0	3	8

In the context of this study, hits and correct rejections were considered true responses, whereas misses and false alarms were considered false responses. Animals were considered proficient in the Shape2Detect task if they met four conditions for two consecutive sessions: (1) at least a 75% accuracy (Eq. 1), (2) 75% precision (Eq. 2), (3) 75% hit rate (Eq. 3) score, and (4) received at least 100 reward pellets.


(1) $$\text{Accuracy}=\frac{\text{Hits}+\text{Correct Rejections}}{\text{Hits}+\text{Misses}+\text{False Alarms}+\text{Correct Rejections}}$$

(2) $$\text{Precision}=\frac{\text{Hits}}{\text{Hits}+\text{False Alarms}}$$

(3) $$\text{Hit}\;\text{Rate}=\frac{\text{Hits}}{\text{Hits}+\text{Misses}}$$

### 2.9. Third tier: detection

Detection was the third tier for the go/no-go task training (Figure 2). The goal of this phase was to maximize animal accuracy during consistently paced trials with invariable parameters. This phase of training was similar to the Shape2Detect task but used fixed behavioral parameters throughout the 60-min-long sessions. These parameters outlined in Figure 3B were the same as those used during the last 20 min of the Shape2Detect sessions (i.e., 6 s trial window duration, 3 s hit window, 50% probability of presenting a stimulus trial, and 8 s timeouts). Animals were considered proficient when they showed at least 75% accuracy, 75% precision, 75% hit rate, 75% correct rejection rate (Eq. 4), and 75% F1-score (Eq. 5) with at least a 1.5 d-prime (*d’*) score (Eq. 6) in three total sessions. The F1-score is a measure of performance in binary classification that considers the harmonic mean, in this case, of an animal’s precision and hit rate scores. The *d’* metric is another performance indicator and common statistical measure used in psychophysical detection tasks and signal detection theory to quantify a subject’s ability to accurately distinguish between a signal and noise within a given task.


(4) $$\text{Correct Rejection Rate}=\frac{\text{Correct Rejections}}{\text{Correct Rejections}+\text{False Alarms}}$$

(5) $$F1\;\text{Score}=2\left(\frac{\text{Precision}*\text{Hit}\;\text{Rate}}{\text{Precision}+\text{Hit}\;\text{Rate}}\right)$$

(6) $$\;d\prime =z\left(\text{Hit}\;\text{Rate}\right)-z\left(\frac{\text{False Alarms}}{\text{False Alarms}+\text{Correct Rejections}}\right)$$

After the training on the go/no-go paradigm was completed, animals underwent five additional Detection sessions to assess baseline accuracy and subject consistency before proceeding to the go/no-go perception threshold detection task.

### 2.10. Go/no-go perception threshold detection task

**Figure 4:**
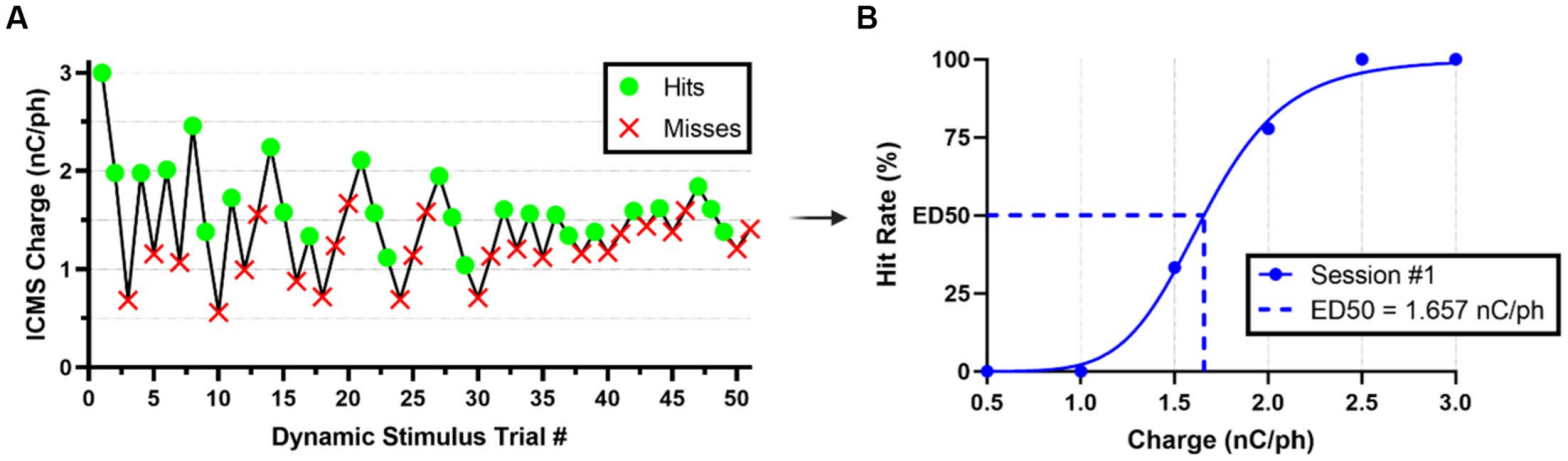
Estimation of ICMS perception thresholds. **(A)** Representative nose-poke response data from the modified staircase presentation of ICMS during a typical threshold detection session. **(B)** Representative quantal dose–response, non-linear regression plot showcasing transformed hit/miss animal response data into percent hit rate based on binned (ranges of 0.5 nC/ph pulsed across all individual channels simultaneously) charge amplitude values presented. Effective charge (dose) at 50% hit rate (ED50) were used to estimate the ICMS perception thresholds.

After rats were fully trained in the go/no-go behavioral paradigm, they were introduced to a dynamic perception threshold detection task that implemented a modified version of the up/down staircase method (Levitt, 1971; Koivuniemi A. et al., 2011), as shown in Supplementary Figure S2. The goal of this task was to approximate an estimation of an animal’s perception threshold value. The first 20 min of every perception threshold detection task began with all ICMS stimulus trials presented at the naïve threshold intensity and with 50% probability (catch trials were presented as the alternative). For the remainder of the session, the naïve threshold intensity was presented with a 33.3% probability, while a dynamic charge intensity was also presented with 33.3% probability (the remainder probability presented a catch trial). The dynamic charge intensities were presented following the modified staircase method (Figure 4A). First, we presented the dynamic charge intensity value at the maximum naïve threshold intensity. If the rat perceived the dynamic charge intensity value and nose-poked, the dynamic charge intensity value was decreased by the step size variation outlined in Table 2. If the rat did not nose-poke, the dynamic charge intensity value was increased. This up/down staircase methodology was followed throughout the session.

**Table 2:** Dynamic stimulus step size variation throughout a 1-h session.

Session time (min)	Step size variation	
	Charge intensity (nC/ph)	Tone intensity (% amplitude)
0–19	No variation	No variation
20–29	1.00 ± 0.40	20.00 ± 5.00
30–39	0.60 ± 0.20	10.00 ± 3.00
40–49	0.40 ± 0.10	1.00 ± 0.30
50–60	0.20 ± 0.05	0.10 ± 0.03

For the auditory stimulus trials, dynamic tone intensity values were determined by modulating the sinusoidal wave amplitude of the training tone. Increases in sinusoidal wave amplitude resulted in a louder and more intensely perceived tone, while decreases produced a quieter and less intense tone. To create a scale for estimating auditory tone thresholds, the amplitude of the training tone was normalized to a range of 0–100%, where 0% represented silence (0 dB SPL) and 100% represented the maximum intensity of the training tone (~90 dB SPL). Similar to the ICMS variation, initial trials in the perception threshold detection task were presented at the maximum training tone intensity of 100% amplitude with a 50% probability. The remaining trials followed the modified staircase method where changes in dynamic tone intensity values were presented to the rats based on their response behavior. Step size variations of auditory tone intensity in percent amplitude are outlined in Table 2.

### 2.11. Estimation of threshold perception

We estimated perception thresholds using non-linear regression (Eq. 7) in a quantal dose–response non-linear regression (Müller and Schmitt, 1990; Liu et al., 2022) in the GraphPad Prism Software ([Agonist] vs. normalized response -- Variable slope, Prism, v9.5.1). In Eq. 7, *x* represents the linear dose in charge/phase or percent amplitude, *y* denotes the normalized response of the percent hit rate from 0 to 100%, and the Hillslope represents the slope factor or steepness of the curve shared globally between all perception threshold detection sessions per animal. We binned the dynamic stimulus trial values into increments of 0.5 nC/ph stimulated across all individual channels simultaneously for the ICMS group and 1% sinusoidal wave amplitude for the auditory group to establish a quantal response (Figure 4B). We defined the effective dose in charge/phase or percent amplitude needed to produce a 50% hit rate response (ED50) as previously demonstrated (Müller and Schmitt, 1990). In this equation, we constrained ED50 so that it must be greater than zero. Finally, perception threshold values were estimated individually for all animals in the ICMS and auditory groups, using the ED50 data collected across five go/no-go perception threshold detection task sessions.


(7) $$\;y=100*\left(x^{\text{HillSlope}}\right)/\left({\text{ED}50}^{\text{HillSlope}}+\left(x^{\text{HillSlope}}\right)\right)$$

### 2.12. Electrochemical characterization

Electrochemical Impedance Spectroscopy (EIS) was conducted before and after the go/no-go perception threshold detection task for comparison as previously described (Joshi-Imre et al., 2019). Briefly, EIS was measured in a three-electrode configuration using a stainless-steel subcutaneous needle (Biopac Systems Inc., CA, USA) as counter and Ag|AgCl disposable dry electrodes (Biopac Systems Inc., CA, USA) as the reference with conducting isotonic electrode gel (Biopac Systems Inc., CA, USA) on the tail of the animal. A 10 mV RMS sinusoidal waveform was applied with respect to the open circuit potential ranging from 10^5^ to 10^0^ Hz using a Reference 600 potentiostat (Gamry Instruments, PA, US). Voltage transients were recorded as previously described (Cogan, 2008; Joshi-Imre et al., 2019) before the go/no-go perception threshold detection task took place, and then again and at the end for comparison. Briefly, the PlexStim stimulator (Plexon, Inc.) was connected and biphasic, cathodal first, current pulses were delivered using the same parameters as those used for ICMS. The PlexStim system has monitor outputs for both the current delivered and the voltage measured. We connected these outputs to an oscilloscope (TBS 1052B, Tektronix, Inc., Beaverton, OR, US) for data collection. Then, we measured the maximum cathodal potential excursion (E_mc_), defined as the electrode potential 12 μs after the end of the cathodal pulse.

### 2.13. Data analysis and statistics

All data analysis was conducted through custom MATLAB (R2022b) scripts, GraphPad Prism (v9.5.1, GraphPad Software, Boston, MA, USA), or Statgraphics Centurion 19 (v19.4.04, Statgraphics Technologies, Inc., The Plains, VA, USA). In MATLAB, we evaluated signal detection theory parameters (Macmillan and Creelman, 2005) for all behavioral sessions, including: accuracy, precision, hit rate, correct rejection rate, F1-score, and *d’* (Eqs 1–6). If a session contained either zero hits, misses, false alarms, or correct rejection responses – all of which are denominators in Eqs 1–6 – then their values were adjusted in order to prevent behavioral performance scores of infinities using a commonly accepted approach (Macmillan and Creelman, 2005). An arbitrary value of 0.5 was added to the metric that had a score of zero (e.g., hits, misses, false alarms, or correct rejections), meanwhile this arbitrary value of 0.5 was subtracted from its non-zero counterpart. For example, if a session contained 119 hits and zero misses, then the adjusted values would be 118.5 hits and 0.5 misses. Then, we generated confusion matrices based on these calculations for each group to highlight the overall accuracies, hit rates, and correct rejection rates during the accuracy baseline Detection task sessions. GraphPad Prism was used to calculate the perception threshold values. Furthermore, we calculated the average training time for each group. For statistical analysis, unpaired two-sample *t*-tests were used to determine significant differences between the ICMS and auditory groups. We conducted a one-tailed paired sample *t*-test between the ICMS results and the intragroup negative control for further validation of this methodology and calculated the post-hoc statistical power using G*Power 3.1 (Faul et al., 2009). We analyzed tests of normality in the data using the Shapiro–Wilk test and confirmed results by examination of their respective QQ plots. Lastly, we performed an equivalence test using Statgraphics Centurion 19 to further investigate if the average ICMS group accuracy was statistically similar or different than the average auditory group accuracy. The upper and lower differential limits were determined from the 95% CI range of the difference between means (Hazra, 2017). Statistical differences of EIS impedance magnitude at 1 kHz and E_mc_ measurements before and after stimulation were calculated using a paired t-test. All results are reported as the mean ± SEM. We defined statistical significance as *p* < 0.05.

## 3. Results

All animals remained above the 90% weekly weight limit for the entire duration of this study, demonstrating that food restriction did not affect their weight. Furthermore, 70% of animals completed the study with at least a 20% increase in overall weight compared to their first shaping session; the remaining animals showed less than 5% weight loss (Supplementary Table S1). All animals passed the Shaping task in less than 10 sessions, resulting in no exclusions from the study due to poor performance.

**Figure 5:**
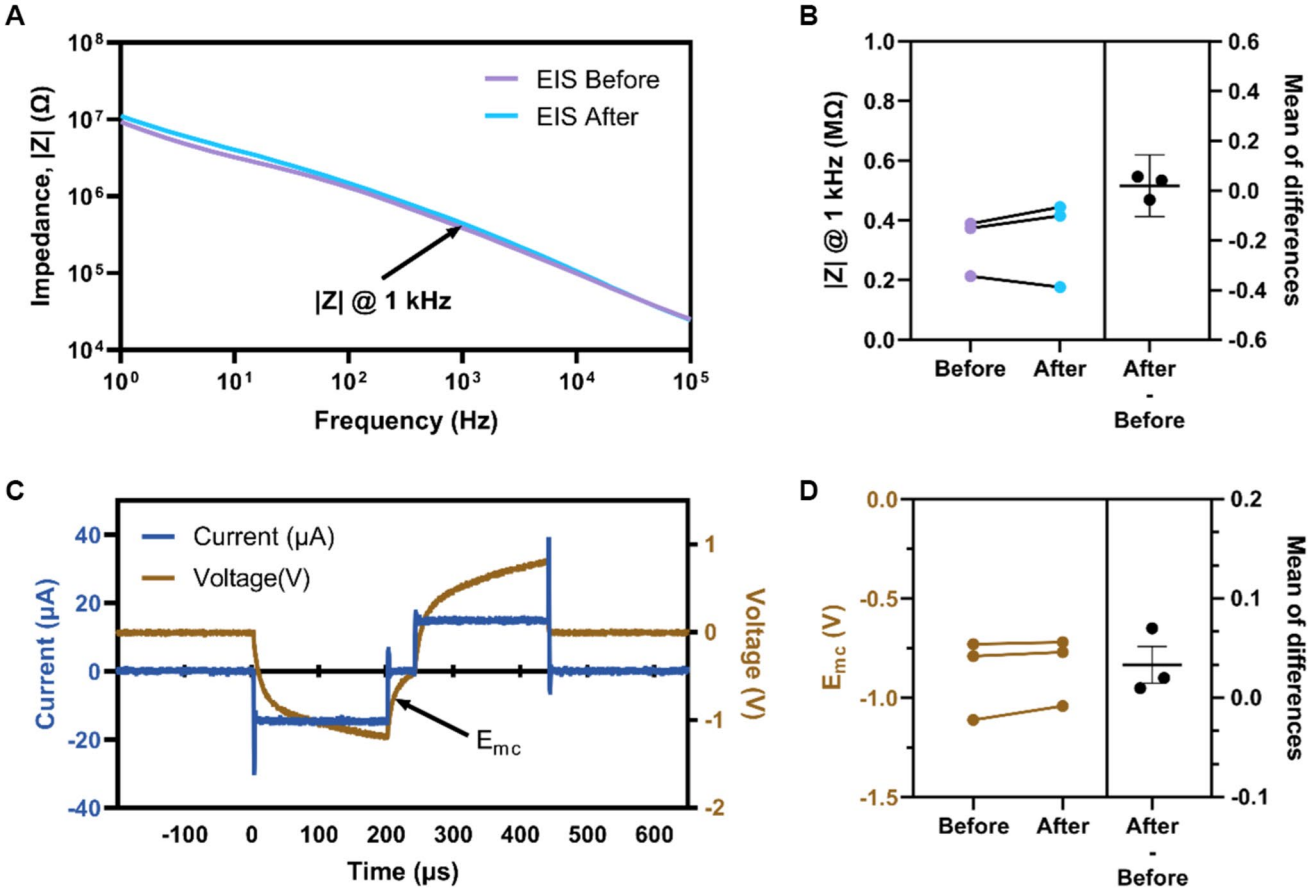
Electrochemical assessment of implanted devices. **(A)** Representative EIS magnitude of a single electrode site before and after stimulation. **(B)** Average impedance magnitude at 1 kHz before and after stimulation for all electrodes per animal. **(C)** Representative voltage transient measurement at 3 nC/ph for an individual channel labeled E_mc_ (arrow). **(D)** Average E_mc_ before and after stimulation for all electrodes per animal.

Animals in the ICMS group were 3.8 ± 0.9 months old at the time of implantation; animals in the auditory group were 3.5 ± 1.1 months old at the time of sham surgery (*p* = 0.30). After implantation of the MEA into the S1FL for animals in the ICMS group, we proceeded with testing of the naïve threshold. All three animals showed a paw withdrawal in the right forepaw, corresponding to the contralateral implant location; two animals responded reliably at 3 nC/ph pulsed across all individual channels simultaneously, and one responded at 4 nC/ph. Voltage transients from each microelectrode array channel were recorded to confirm set stimulation parameters outlined within the Electrical Stimulation and Auditory Parameters subsection. Figure 5A shows representative EIS for a single electrode before and after the go/no-go task. Quantification of the impedance magnitude at 1 kHz (Figure 5B) shows that the difference between before (325.20 ± 56.29 kΩ) and after (345.20 ± 85.09 kΩ) stimulation was not statistically significant (*p* = 0.56). Figure 5C shows a representative *in-vivo* voltage-transient measurement of the current-controlled pulse with an amplitude of 15 μA (30 nC/ph) for a single channel. We found that the E_mc_ before stimulation was −0.88 ± 0.12 V and after stimulation was −0.84 ± 0.09 V (Figure 5D). There was no statistical difference (−0.03 ± 0.01 V; *p* = 0.21) between the measured E_mc_ before and after stimulation. Electrochemical assessment showed that the electrodes delivered electrical stimulation consistently and remained unchanged throughout the sessions, validating that the applied current amplitude was delivered as set in the MATLAB custom GUI.

### 3.1. Go/no-go behavioral training

**Figure 6:**
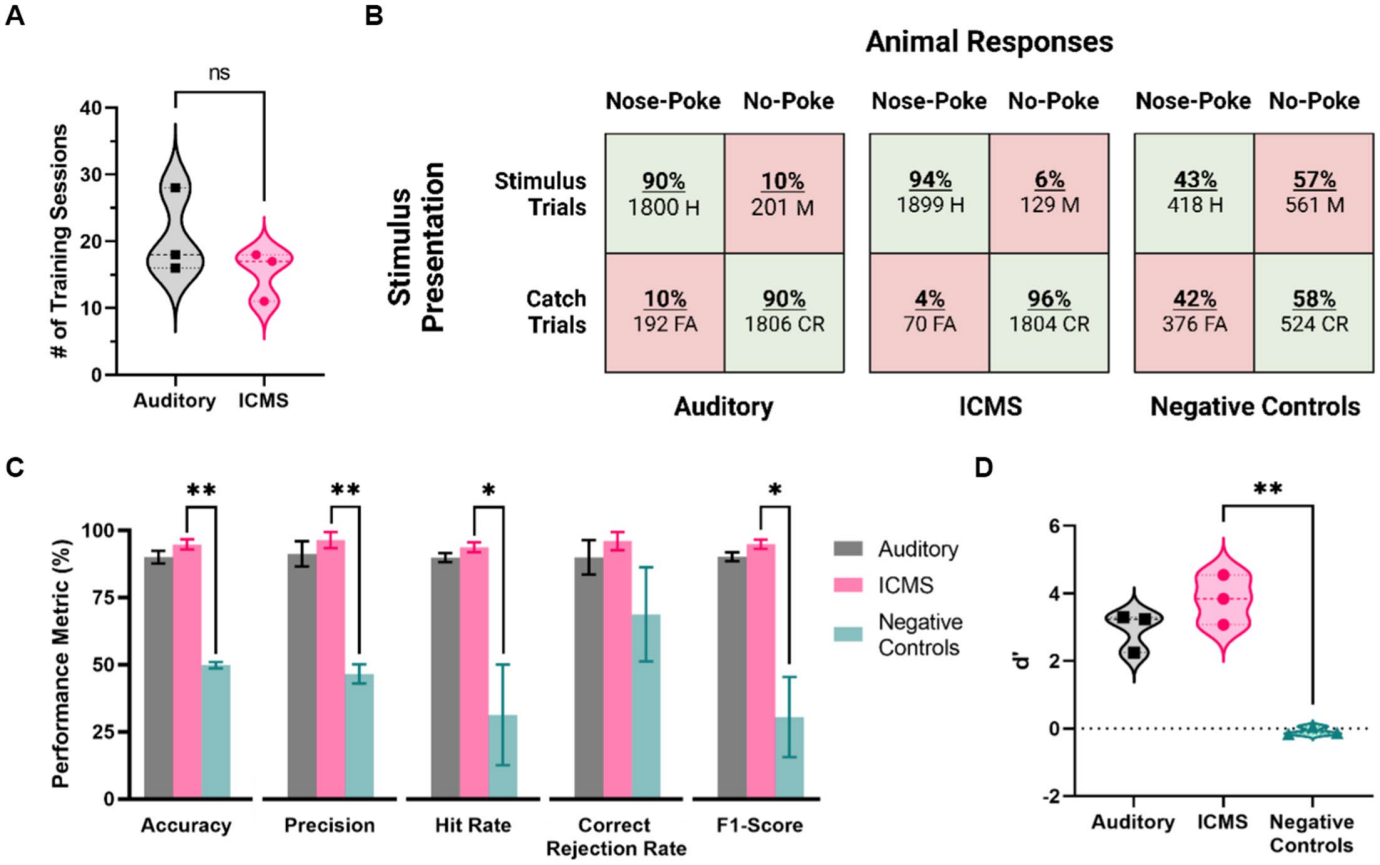
Behavioral performance metrics for the ICMS and auditory groups, and for negative control stimulation. **(A)** Training time for each group, in number of sessions needed to pass the training phase. **(B)** Confusion matrices showing presented trials (rows) and animal responses (columns). Values depict all animal response data from five baseline accuracy sessions. **(C)** Behavioral performance metrics, including accuracy, precision, hit rate, correct rejection rate, and F1-Score. Data are shown as mean ± SEM. **(D)** Average scores of the *d’* metric. *p<0.05 and **p<0.01.

Figure 6 provides the assessment of behavioral proficiency in the go/no-go task. As shown in Figure 6A, animals in the ICMS group took an average of 15.3 ± 2.2 sessions in total between Shaping, Shaping2Detect and Detection tasks, while animals in the auditory group took an average of 20.7 ± 3.7 sessions (*p* = 0.28). This number of sessions corresponds to 4–5 weeks of training for the animal to become proficient in the go/no-go behavioral task.

Then, we proceeded to assess the baseline performance on the go/no-go behavioral task of each animal in five post-training sessions. Figure 6B shows the overall distribution of the total presented trials (rows) and animal responses (columns) for each group, represented in the form of confusion matrices. There was a total of 3,902 trials presented for the ICMS animals, including stimulus (2,028) at the naïve threshold and catch (1,874) trials. In comparison, the auditory group received 3,999 total trials (stimulus trials: 2,001, catch trials: 1,998). Animals in both, auditory and ICMS groups showed similar hit rates (auditory = 90%, ICMS = 94%), showing that the animals are correctly poking upon most stimulation trials. Similarly, animals in both groups had a high correct rejection rate (auditory = 90%, ICMS = 96%). These results indicate that both groups of animals were able to greatly recognize a stimulus signal and respond with a nose-poke. In contrast, when the stimulation was turned off for the ICMS group (negative control) the hit rate dropped down to only 43% and correct rejections to only 58%, signifying random poking. Figure 6C outlines the accuracy performance metrics for all groups. The average accuracy scores between the ICMS (94.7 ± 1.9%) and auditory (90.0 ± 2.4%) groups were comparable to one another (*p* = 0.19). In addition, the equivalence test performed subsequently demonstrated that the accuracy for both groups was equivalent (*p* = 0.03). In contrast, the ICMS and negative controls (49.8 ± 1.2%) were significantly different for accuracy (*p* = 0.002). The statistical power was found to be 96.03% with an ICMS effect size of 3.31. In addition, two of the auditory group animals completed three *post hoc* negative control sessions, showing an average accuracy score of 46.17%, comparable to that of the ICMS negative controls (*p* = 0.34). The average precision scores between the ICMS (96.4 ± 3.0%) and auditory (91.2 ± 4.7%) groups were comparable (*p* = 0.41); the difference between ICMS and negative controls (46.6 ± 3.6%) was statistically significant (*p* = 0.008). The average hit rates between the ICMS (93.7 ± 1.8%) and auditory (89.9 ± 1.7%) groups comparable (p = 0.19); differences between the ICMS group and negative controls (31.3 ± 18.8%) were statistically significant (*p* = 0.04). The average correct rejection rates between the ICMS (96.0 ± 3.3%) and auditory (89.9 ± 6.4%) groups were comparable (*p* = 0.45); difference between ICMS and negative controls (68.7 ± 17.6%) did not reach statistical significance (*p* = 0.10). These correct rejection rates show that all animals were able to identify catch trials regardless of stimuli type.

The average F1-scores between the ICMS (94.9 ± 1.7%) and auditory (90.2 ± 1.7%) groups were comparable (*p* = 0.12). The difference between the ICMS and negative controls (30.5 ± 14.9%) was statistically significant (*p* = 0.03), further demonstrating that animals are only poking upon stimulus presentation. In addition, the average *d*’ scores (Figure 6D) between the ICMS (3.82 ± 0.43) and auditory (2.93 ± 0.34) groups were comparable (*p* = 0.18); the difference between ICMS and negative controls (−0.08 ± 0.07) was found to be statistically significant (*p* = 0.008), demonstrating that the animals are able to distinguish between stimulus and catch trials.

### 3.2. Estimated perception thresholds

**Figure 7:**
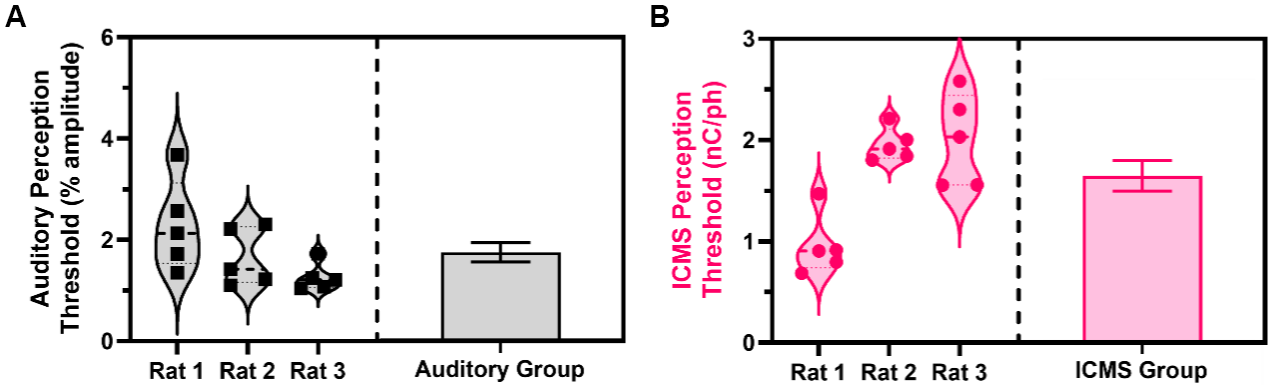
Estimated perception thresholds for the ICMS and auditory animal groups. **(A)** Estimated perception threshold values plotted for each auditory animal (left) and auditory group estimations (right) shown as mean ± SEM. **(B)** Estimated perception threshold values plotted for each ICMS animal (left) and ICMS group estimations (right) shown as mean ± SEM.

Across five sessions of the go/no-go perception threshold detection task, we estimated the perception thresholds for all animals in the auditory and ICMS groups. Figure 7A (left) shows the estimated perception threshold values for individual sessions for each animal in the auditory group. The perception threshold between sessions for each animal showed a standard deviation from the mean ranging from 0.27 to 0.90% of the sinusoidal wave amplitude. Figure 7A (right) shows the summary statistics, where the perception threshold was estimated at 1.74 ± 0.19% sinusoidal wave amplitude. Figure 7B (left) shows the estimated perception threshold values for individual sessions for each animal. Animals in the ICMS group showed a small standard deviation from the mean ranging from 0.16 to 0.45 nC/ph pulsed across all individual channels simultaneously in the perception thresholds across all five sessions. Figure 7B (right) shows that the average perception threshold across all animals is 1.64 ± 0.15 nC/ph pulsed across all individual channels simultaneously.

## 4. Discussion

In this study, we developed and validated an innovative non-pain aversive, go/no-go behavioral paradigm based on a nose-poking task to quantify rat sensory perception thresholds in response to ICMS. Our results showed that this nose-poking paradigm could reliably assess stimulation-evoked sensory percepts in rats originating from ICMS in the S1FL and its accuracy was comparable to the well-established auditory discrimination task.

The study of auditory tone discrimination tasks in animals has a long and rich history in neuroscience research. Early studies in the 1970s focused on fundamental aspects of auditory perception in rats, such as their ability to detect pure tones and discriminate between tones of different frequencies and intensities (Kelly and Masterton, 1977). These studies laid the foundation for more complex auditory tasks developed in the following decades (Hui et al., 2009; Sloan et al., 2009). One such task is the go/no-go task, which once involved training rats to press a lever in response to a specific tone (the “go” tone) and withhold their response to other tones (“no-go” tones) (Engineer et al., 2008). Then, this go/no-go task was modified from lever-pressing to nose-poking because it was found to require less experimenter intervention for a naïve rat to reliably perform the task with the addition of a higher baseline rate of responding and lower between-group variability (Mekarski, 1988; Schindler et al., 1993). This nose-poke go/no-go behavioral paradigm has been used by multiple research groups and is widely accepted because of its straightforwardness to train rats with nose-poking being an innate exploration behavior, the hardware is available off-the-shelf and does not require complex motors and controls, and it has shown high accuracy rates of up to ~90% (Sloan et al., 2009; Riley et al., 2021). Overall, the history of auditory tone discrimination tasks in rats highlights their broad utility as a model system for studying auditory perception and processing. For the development of the behavioral paradigm presented here, we built upon this nose-poke-based, go/no-go paradigm.

To validate the presented behavioral paradigm, we compared the ICMS group to an auditory discrimination control group. Using the auditory discrimination group as positive controls allowed us to establish an effective baseline to compare accuracy and reliability of our behavioral paradigm. Within our study, the auditory control group showed an accuracy of ~90% and demonstrated an auditory tone threshold of approximately 2% amplitude (~65 dB SPL), which is comparable to previous literature (Engineer et al., 2008; Sloan et al., 2009; Riley et al., 2021). These results validate our implementation of the nose-poke behavioral paradigm, and our method of using non-linear regression for estimating threshold perception. The ICMS group had a comparable accuracy to the auditory control group of ~95%. Although the ICMS group appeared to have a higher accuracy score than the auditory group, there were no statistically significant differences between groups. Furthermore, the *post hoc* equivalence test on the accuracy scores provided evidence that both groups performed comparably, which validates the use of this go/no-go nose-poke task for the assessment of ICMS perception. Furthermore, animals in both groups underwent a negative control phase at the end of the study to confirm that the nose-poking behavior was neither random nor were the animals nose-poking on any confounding cues. Results from this second phase of the investigation yielded an accuracy of less than 50%, which is an indication of random poking, further validating with the present methodology.

The measurement of naïve thresholds shortly after implantation provided us with a threshold known to evoke a sensory percept for each animal, which was then used for training. These naïve thresholds ranged between animals from 3 to 4 nC/ph. We believe that this variability may be attributed to micron-scale shifts in implant location, which may have resulted in somatotopic differences between animals. Then, using the validated quantal non-linear regression at the ED50 level, we established that the average electrical perception threshold across three animals was approximately 1.64 nC/ph pulsed across all 10 individual channels simultaneously with the lowest animal averaging 0.96 nC/ph. Previous animal behavioral paradigms have been developed to study sensory and visual perception via ICMS, including rodents, cats, non-human primates, and humans (Tehovnik, 1996; Rousche and Normann, 1999; Ni and Maunsell, 2010; Fernández et al., 2021; Lycke et al., 2023), which have identified different thresholds of perception. Urdaneta et al. (2022) demonstrated perception thresholds ranging between 6.4 and 10.7 nC/ph for rat cortex, when stimulating Ir electrode sites individually. The same group has demonstrated that delivering electrical stimulation through two or more electrode sites simultaneously can reduce the perception threshold (Kunigk et al., 2022) by at least 53% of the single site perception threshold. Other studies have shown lower perception thresholds using traditional microelectrode arrays in cat somatosensory cortex (Rousche and Normann, 1999) with an approximate threshold of 1.5 nC/ph; non-human primates between 1 and 2 nC/ph (Ni and Maunsell, 2010; Callier et al., 2015; Ferroni et al., 2017); and human studies ranging from 0.4 to 3 nC/ph (Schmidt et al., 1996; Flesher et al., 2016; Fernández et al., 2021; Hughes et al., 2021). A different study targeting the primary somatosensory cortex in mice (Lycke et al., 2023) found the lowest perception threshold of 0.25 nC/ph stimulating individual and multiple electrode sites simultaneously. It should be noted that stimulation parameters, MEAs, implantation targets, and number of electrode sites pulsed are not consistent between these studies. Nevertheless, results from these prior studies demonstrate broad consistency with the estimated perception thresholds in the present work.

Some Institutional Animal Care and Use Committees (IACUCs) require *ad libitum* access to water for a minimum of 1 h for at least every 12 h, which may further limit the deployment of previous water-restrictive behavioral paradigms to other research groups. Food restriction is preferred over water restriction by most IACUCs. In this paradigm we mildly restricted food intake, an approach ethically preferred over water deprivation, to ensure rodent engagement during the behavioral task. At the end of each session, animals were given supplemental feed to ensure appropriate nutrition. However, both water deprivation and food restriction have been associated with a stress response characterized by an upregulation of adrenal corticosterone (Dietze et al., 2016; Vasilev et al., 2021). It is unknown whether this stress response may play a role in the reliability of intracortical MEAs and stability of ICMS. To address this concern, we food-deprived the animals so that their weight would not fall below 90% of their initial weekly weight and fed supplemental nutrition whenever necessary to prevent weight loss and support growth. This ensured animals’ welfare and demonstrated growth for most of them; two animals showed weight decrease that was found to be less than the 10% threshold for our established protocol. This approach has been widely validated in nose-poke rodent behavioral tasks that rely on food deprivation while still promoting high accuracy scores (Engineer et al., 2008; Riley et al., 2021). Future work may consider methods to avoid food restriction while participating in the nose-poke task.

A final limitation of this study was the training time, resulting from having a mostly positive reinforcement behavioral task. Animals in this study underwent 1 week of Shaping, 3–4 weeks of Shape2Detect, 1–2 weeks of Detection and 1 week of the accuracy baseline Detection task assessment for a total of 6–8 weeks of training. During this time, we could not assess perception thresholds, meaning that we could not assess changes during the first 6–8 weeks post-implantation. Previous studies (Urdaneta et al., 2022) have reported training phases of up to 8 weeks post implantation, comparable to the number of sessions required for training in the present paradigm. However, this acute phase is known for presenting changes to the MEA surrounding tissues, including myelin degeneration and glial encapsulation. Assessment during the acute phase would provide information regarding perception threshold and documented tissue response. In future studies, we will optimize the training time to assess perception thresholds as early as possible after implantation by increasing the probability of presenting a stimulus trial during the Shape2Detect and Detection phases of training and lowering the threshold to pass from one training stage to the next.

Despite these limitations, this study presents an effective behavioral paradigm for evaluating ICMS-evoked somatosensory percepts in rats. However, there are still known challenges associated with rat ICMS studies apart from establishing a reliable perception threshold indicator. For example, it has been well-documented that perception thresholds change over time (Koivuniemi A. et al., 2011; Callier et al., 2015; Hughes et al., 2021; Bjånes et al., 2022; Kunigk et al., 2022; Lycke et al., 2023). In the future we will employ this behavioral paradigm to study ICMS-evoked perception threshold stability of novel MEA device technologies that aim at improving the long-term reliability of the neural interface. Finally, the control software that we have developed for this paradigm is open-source and available to download at no cost. This will allow research groups who are interested in evaluating long-term stability of novel stimulating MEAs (especially those whose IACUC prefer food restriction over water deprivation in rodents) to easily adopt this go/no-go behavioral paradigm using hardware available off-the-shelf.

## 5. Conclusion

In this study we presented a new, highly accurate behavioral paradigm to assess ICMS-evoked somatosensory perception thresholds. This paradigm builds upon well-established and accepted auditory discrimination tasks with comparable results, validating the go/no-go behavioral task for the assessment of ICMS-evoked percepts. Full deployment of this paradigm establishes a new platform for elucidating the information processing principles in the neural circuits related to neuroprosthetic sensory perception and for studying the performance of novel MEA device technologies using freely moving rats. Future studies will assess how MEA design and cortical circuitry impacts stimulus response-time circuitry, threshold sensitivity, and selectivity discrimination for the primary somatosensory cortex.

## Data availability statement

The raw data supporting the conclusions of this article will be made available by the authors, without undue reservation.

## Ethics statement

The animal study was reviewed and approved by The University of Texas at Dallas Institutional Animal Care and Review Committee.

## Author contributions

TS: conceptualization, methodology, software, validation, formal analysis, investigation, resources, writing – original draft, visualization, and project administration. YW: investigation. CC: investigation. AK: investigation. HS: investigation. JC: conceptualization, writing – review and editing, and funding acquisition. SC: conceptualization, resources, writing – review and editing, and funding acquisition. JP: conceptualization, resources, writing – review and editing, project administration, and funding acquisition. CE: methodology, resources, and writing – review and editing. AH-R: conceptualization, methodology, software, formal analysis, writing – review and editing, and project administration. All authors contributed to the article and approved the submitted version.

## Funding

This work was supported in part by the National Institutes of Health, National Institute for Neurological Disorders and Stroke (R01NS110823, GRANT12635723, JC and JP), diversity supplement to parent grant (AH-R), a Research Career Scientist Award (GRANT12635707, JC) from the United States (US) Department of Veterans Affairs Rehabilitation Research and Development Service, and the Eugene McDermott Graduate Fellowship from The University of Texas at Dallas (202108, TS).

## Conflict of interest

CE was married to an employee of Microtransponder, Inc., a company that develops vagus nerve stimulation therapies. Microtransponder was not involved in the development or analysis of this research.

The remaining authors declare that the research was conducted in the absence of any commercial or financial relationships that could be construed as a potential conflict of interest.

## Publisher’s note

All claims expressed in this article are solely those of the authors and do not necessarily represent those of their affiliated organizations, or those of the publisher, the editors and the reviewers. Any product that may be evaluated in this article, or claim that may be made by its manufacturer, is not guaranteed or endorsed by the publisher.

## Footnotes

^1^ https://github.com/Neuronal-Networks-and-Interfaces-Lab/Stimulation-Evoked_Perception_Behavioral_Software.git
