# Supplementary material for: Behavioral paradigm for the evaluation of stimulation-evoked somatosensory perception thresholds in rats
Source: Front Neurosci. 2023 Jun 13;17:1202258. doi: 10.3389/fnins.2023.1202258 (PMC10293669; doi:10.3389/fnins.2023.1202258)
Supplement: Supplementary file 1 [file Data_Sheet_1.PDF]

frontiers

Supplementary Material

Behavioral Paradigm for the Evaluation of Stimulation-Evoked Somatosensory Perception Thresholds in Rats

Thomas J. Smith [1], Yupeng Wu [2], Claire Cheon [3], Arlin A. Khan [1], Hari Srinivasan [1], Jeffrey R. Capadona [4,5], Stuart F. Cogan [3], Joseph J. Pancrazio [3], Crystal T. Engineer [1,6], Ana G. Hernandez-Reynoso [3,*]

[1] School of Behavioral and Brain Sciences, The University of Texas at Dallas, Richardson, TX, United States

[2] Department of Materials Science and Engineering, The University of Texas at Dallas, Richardson, TX, United States

[3] Department of Bioengineering, The University of Texas at Dallas, Richardson, TX, United States

[4] Department of Biomedical Engineering, Case Western Reserve University, Cleveland, OH, United States

[5] Advanced Platform Technology Center, Luis Stokes Cleveland Veterans Affairs Medical Center, Cleveland, OH, United Sates

[6] Texas Biomedical Device Center, The University of Texas at Dallas, Richardson, TX, United States

*** Correspondence:**
Ana G. Hernandez-Reynoso:
ana.hernandezreynoso@utdallas.edu

1 Supplementary Figures and Tables

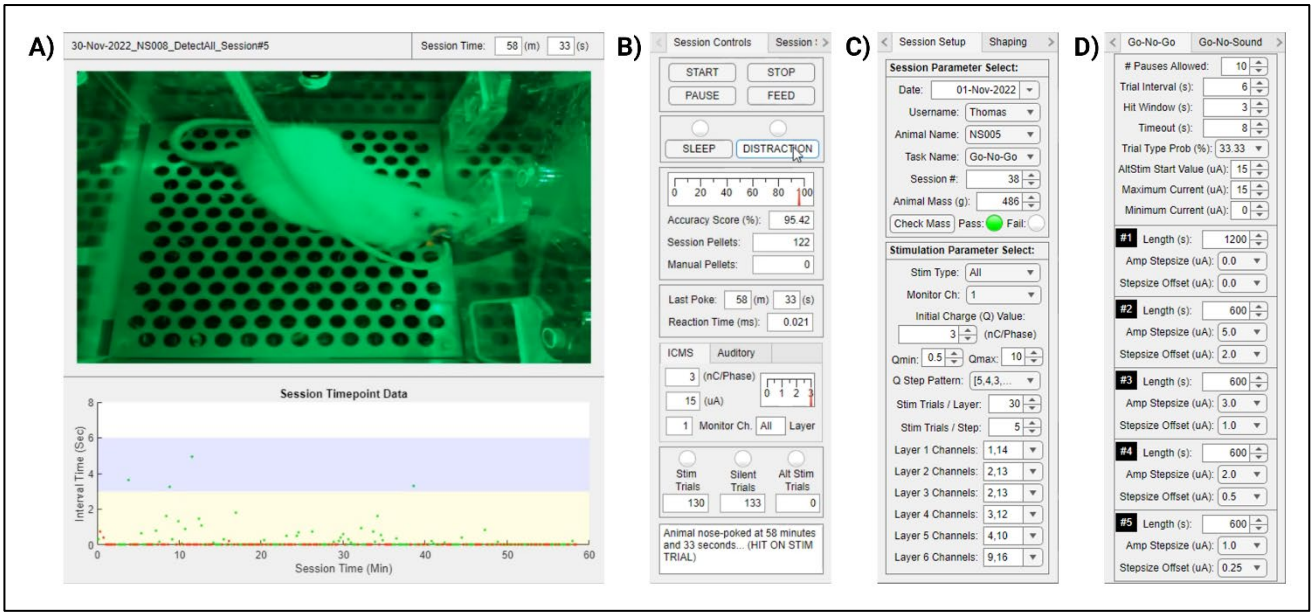

Supplementary Figure 1. Custom MATLAB GUI application. (A) Main screen showing live video feed (top) from behavioral chamber during a stimulation trial, and nose-poke response data (bottom) throughout the session. **(B)** Session controls showing buttons to start, pause, and stop the session manually, and to manually feed a reward pellet. In addition, there are two buttons to mark times during the session when the animal is distracted or sleeping. The remainder of the panel displays live performance and behavioral task metrics such as session accuracy, number of reward pellets eaten, timepoint of last nose-poke, trial reaction time, stimulus intensity values, and a text box that presents various status updates. **(C)** Session setup panel displaying options for selecting the date, researcher, animal, task name, current session number and a button to confirm animal mass at or above 90% free feeding level. It also contains an ICMS parameter selection panel used to define the intensity of the stimulus and electrode channels used. **(D)** Example behavioral task panel showing the options for changing the go/no-go task parameters outlined in the study.

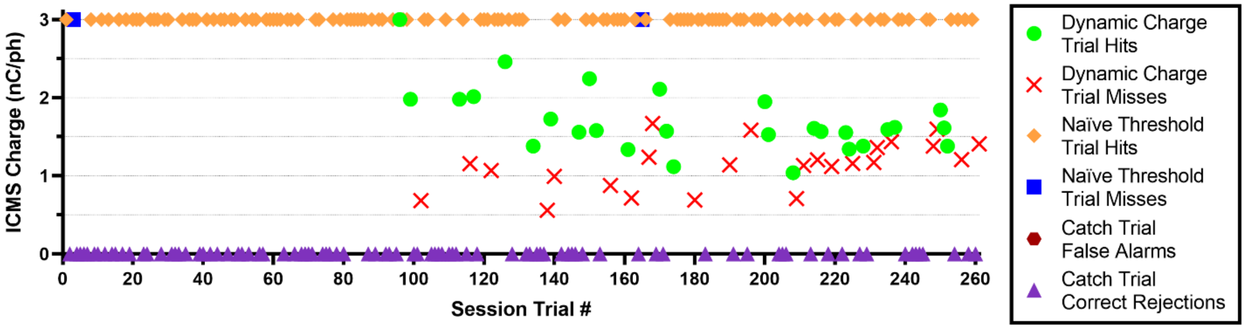

Supplementary Figure 2. Perception Threshold Detection Task session showing all trial response types. (A) Representative animal response data from the go/no-go perception threshold detection task session. This chart plots an ICMS animal's responses in hits or misses for the dynamic stimulus and naïve stimulus trials, and false alarms or correct rejections for the catch trials presented throughout a typical one-hour session. There were no catch trial false alarms present within this example session. Additionally, the dynamic charge trial hits and misses plotted here are equivalent to the hits and misses plotted in Figure 4A.

Supplementary Table I. Animal Weight Progression

		Session Weight		
**Animal Group**	**Animal**	**First Session (g)**	**Last Session (g)**	**% Change**
ICMS	Rat 1	412	578	40.29
ICMS	Rat 2	355	517	45.63
ICMS	Rat 3	522	503	-3.64
Auditory	Rat 1	452	569	25.88
Auditory	Rat 2	550	523	-4.91
Auditory	Rat 3	362	436	20.44
